# Identifying Susceptibility Loci for Cutaneous Squamous Cell Carcinoma Using a Fast Sequence Kernel Association Test

**DOI:** 10.3389/fgene.2021.657499

**Published:** 2021-05-10

**Authors:** Manyan Huang, Chen Lyu, Xin Li, Abrar A. Qureshi, Jiali Han, Ming Li

**Affiliations:** ^1^Department of Epidemiology and Biostatistics, School of Public Health, Indiana University at Bloomington, Bloomington, IN, United States; ^2^Department of Epidemiology, Richard M. Fairbanks School of Public Health, Indiana University – Purdue University Indianapolis, Indianapolis, IN, United States; ^3^Melvin and Bren Simon Cancer Center, Indianapolis, IN, United States; ^4^Department of Dermatology, Alpert Medical School, Brown University, Providence, RI, United States

**Keywords:** region-based association test, fast sequence kernel association test, cutaneous squamous cell carcinoma, rare variants, generalized genetic random field

## Abstract

Cutaneous squamous cell carcinoma (cSCC) accounts for about 20% of all skin cancers, the most common type of malignancy in the United States. Genome-wide association studies (GWAS) have successfully identified multiple genetic variants associated with the risk of cSCC. Most of these studies were single-locus-based, testing genetic variants one-at-a-time. In this article, we performed gene-based association tests to evaluate the joint effect of multiple variants, especially rare variants, on the risk of cSCC by using a fast sequence kernel association test (fastSKAT). The study included 1,710 cSCC cases and 24,304 cancer-free controls from the Nurses’ Health Study, the Nurses’ Health Study II and the Health Professionals Follow-up Study. We used UCSC Genome Browser to define gene units as candidate loci, and further evaluated the association between all variants within each gene unit and disease outcome. Four genes *HP1BP3, DAG1, SEPT7P2*, and *SLFN12* were identified using Bonferroni adjusted significance level. Our study is complementary to the existing GWASs, and our findings may provide additional insights into the etiology of cSCC. Further studies are needed to validate these findings.

## Introduction

Cutaneous squamous cell carcinoma (cSCC) is the second most common type of non-melanoma skin cancers, accounting for about 20% of all skin cancers and the majority of deaths attributable to non-melanoma skin cancers (Chitsazzadeh et al., 2016; Motaparthi et al., 2017; Parekh and Seykora, 2017; Que et al., 2018a). The incidence of cSCC in the United States has been increasing over the last few decades, with over 1 million annual cases in recent years (Nguyen et al., 2014; Muzic et al., 2017; Que et al., 2018a,b). The increase is also expected to continue because of the longer life expectancy, aging population and chronic ultraviolet exposure (Nguyen et al., 2014; Motaparthi et al., 2017; Waldman and Schmults, 2019). The growing mortality and morbidity of cSCC has posed immense economic burden on the national healthcare systems. Though the remission rate of cSCC cases has substantially improved, many cases were still associated with higher probability of recurrence, metastasis and poor prognosis after surgery (Motaparthi et al., 2017; Que et al., 2018a; Waldman and Schmults, 2019). It is of crucial importance to understand the pathogenesis of cSCC and to reduce the public health impact of the disease.

The etiology of cSCC has not been fully understood. However, the risk of the disease can be influenced by multiple environmental exposures. For example, higher risk of cSCC is found to be associated with increased age, fair skin color, male gender, exposure to ultraviolet radiation, immunosuppression and human papillomavirus (Chahal et al., 2016; Parekh and Seykora, 2017; Que et al., 2018a; Waldman and Schmults, 2019). Similar to all cancers, genetic susceptibility also plays an important role in the development of cSCC. Familial aggregation provides direct evidence for the heritability of cSCC (Hussain et al., 2009; Asgari et al., 2015). A few known cancer-related genes, such as *TP53, CDKN2A*, *Ras*, and *NOTCH1* were also causal to skin cancers (Que et al., 2018a). Mutations with these genes may disrupt normal cell growth, cell circle and cellular signal transduction, leading to the development of the disease. In the past decade, genome-wide association studies (GWAS) have become a commonly used strategy to identify genetic variants for complex human diseases in the general population. A few GWASs have identified multiple genetic variants that are associated with the risk of cSCC, such as *CADM1, AHR, SEC16A*, and *DEF8* (Nan et al., 2011; Asgari et al., 2016; Chahal et al., 2016; Siiskonen et al., 2016). Many findings were also successfully replicated in independent populations. These findings have provided valuable insights into the genetic etiology of cSCC.

Despite of these successes, it was estimated that the genetic variants identified by existing GWASs only account for ∼8.5% of the cSCC heritability (Sarin et al., 2020). The genetic causes of the disease remain largely unknown (Chahal et al., 2016). This challenge may be due to a number of limitations of the existing GWASs, such as insufficient statistical power to detect small to moderate genetic effects, burden of multiple testing adjustment, and overlooking potential interactions among variants (Mo et al., 2015; Nettiksimmons et al., 2016). As an alternative to the single-locus analysis, gene- or region-based analysis can be a complementary approach addressing some of those limitations. It may integrate effects of multiple genetic variants, especially rare variants, within a genetic region for improved power, reduce the computational intensities and alleviate the burden of multiple testing (Wu et al., 2010). In recent years, a number of statistical methods have been developed for conducting region-based association test. For example, a sequence kernel association test (SKAT) has been a commonly used method that evaluates the joint effects of genetic variants in a region on a disease outcome while adjusting for covariates (Wu et al., 2011). It uses flexible kernel functions to integrate the effects from multiple variants and allows the effect of causal variants to be bi-directional. Further, a fast sequencing kernel association test (fastSKAT) has been developed to implement SKAT in a computational efficient fashion, especially for large-scale studies with thousands of subjects (Lumley et al., 2018). In this article, we assessed the validity of region-based fastSKAT by replicating 18 GWAS-identified SNPs using single-locus testing. We further tested the association between approximately 23,000 gene regions and cSCC outcome in five independent study populations. The results from each population were further integrated by a Fisher’s combined probability test.

## Materials and Methods

### Ethics Statement

The study protocol was approved by the institutional review boards of the Brigham and Women’s Hospital and Harvard T.H. Chan School of Public Health, and those of participating registries as required.

### Study Population

Our study included 26,014 individuals from three large prospective cohort studies in the U.S., including the Nurses’ Health Study (NHS), the Nurses’ Health Study 2 (NHS2), and the Health Professionals Follow-up Study (HPFS). The subjects were selected under a nested case-control design based on cSCC status. Cases were defined as individuals diagnosed with invasive cSCC, while controls were individuals free of cSCC or any primary type of cancers. The individuals’ characteristics, genotypes and other covariates information were collected in the NHS, the NHS2 and the HPFS studies. In this study, we partitioned the subjects into five independent sub-populations based on their genotyping platforms, including “Affymetrix,” “Illumina,” “OmniExpress,” “OncoArray” and “HumanCore.” In the following, we used these platforms to represent five populations. After the quality control process, the five populations included a total of 5,533, 3,314, 5,354, 5,267, and 6,646 subjects, respectively. More details about the study design and data collection were described elsewhere (Chahal et al., 2016; Duffy et al., 2018).

### Genomic Imputation and Quality Control

The genomic datasets, imputation and quality control procedures were conducted separately in each population and were described with details in previous publications (Lindström et al., 2017; Duffy et al., 2018). Briefly, the participants from five sub-populations were genotyped at different times and by different genotyping platforms. The subjects in “Affymetrix” were genotyped by the Genome-wide Human SNP Array 6.0. The subjects in “Illumina” were genotyped by either Illumina HumanHap300 BeadChip, HumanHap550-Quad BeadChip, Human610-Quad BeadChip, or Human660W-Quad BeadChip. The subjects in “OmniExpress” were genotyped by Illumina HumanOmniExpress-12 BeadChip. The subjects in “OncoArray” were genotyped by Infinium OncoArray-550K BeadChip. The subjects in “HumanCore” were genotyped by Illumina HumanCoreExome-12v1-0 BeadChip.

Variants with low call rate (<95%) were removed. A pairwise identity-by-descent (IBD) analysis was conducted to identify duplicates. For individuals who may be genotyped for more than once using different genotyping platforms, one of the duplicated pair was excluded by the order of “Affymetrix,” “Illumina,” “OmniExpress,” “OncoArray,” and “HumanCore.” For individuals with different cohort IDs but a high genetic concordance rate, both of the pairs were removed. Genome imputation was further conducted in each population using the 1000 Genomes Project Phase 3 Integrated Release Version 5 as reference panels. Software *ShapeIT* (v2.r837) was used for genotype phasing, and the phased genotypes were further imputed to ∼ 47 million variants using *Minimac3* (O’Connell et al., 2014; Das et al., 2016).

### Replication of GWAS Identified SNPs Using Single-Locus Testing

To evaluate the validity of fastSKAT, we used 18 SNPs identified in two previous GWAS as positive controls (Chahal et al., 2016; Sarin et al., 2020). In these previous GWASs, ten SNPs were identified involving 3 independent populations (i.e., “Affymetrix,” “Illumina,” and “OmniExpress”), and 8 SNPs were identified using all 5 populations. For comparison purpose, we first used fastSKAT to test the association between each of these SNPs and cSCC, and further conducted a Fisher’s combined probability test to evaluate the overall association across three or five populations consist with their analysis in the previous GWASs. For fair comparison, we calculated *p*-values by applying fastSKAT to the same NHS and HPFS populations used in previous publications. In particular, “Affymetrix,” “Illumina,” and “OmniExpress” were used in Chahal et al. (2016), while “Affymetrix,” “Illumina,” “OmniExpress,” “OncoArray,” and “HumanCore” were all used in Sarin et al. (2020). The *p*-values were compared to those of previous GWAS publications for consistency.

### Genomic Region Selection

To identify biologically meaningful loci, we used UCSC Genome Browser (assembly GRCh37/hg19) to define gene units as candidate loci for region-based analysis. Software *bedtools* were used to merge the redundant and overlapping genomic regions based on the gene annotation (Kindlon ARQaN, 2009–2019; Quinlan and Hall, 2010). A candidate locus was then defined as 7.5KB upstream and downstream the corresponding gene region. Ultimately, a total of 25,437 regions were extracted. During the data processing, SNPs with an imputation quality score less than 0.3 were removed. We also extracted common and rare variants separately for each region using *PLINK2.0* (Purcell et al., 2007; Purcell). Common and rare variants were defined based on whether the minor allele frequency (MAF) was larger than 5%. Because previous GWAS has comprehensively evaluated each single variant for association with cSCC, we only considered regions with two or more variants for region-based association analysis.

### Region-Based Association Test

We evaluated the association between genomic regions and cSCC using the fastSKAT, a region-based association test that is computationally efficient for large-scale genomic datasets (Lumley et al., 2018). Similar to the SKAT method, it is a variance component score test that integrates the effect of multiple genetic variants within the same region (Wu et al., 2011). The improvement of computational speed over SKAT was achieved by accurately approximating the tail probability for the asymptotic distribution of the test statistics (Lumley et al., 2018). Instead of computing all the eigenvalues of the genotypic similarity matrix, only the top ones were computed through random projections (Halko et al., 2011; Tropp, 2011). The tail probability can then be approximated by the top eigenvalues and a reminder term computed using Satterthwaite approximation, which approximates the sum of weighted chi-square distributions with a single chi-square distribution. The fastSKAT has been implemented in R package “bigQF” (Lumley et al., 2018). For each gene region, the method was applied for rare variants (MAF < 5%) and common (MAF ≥ 5%) variants separately, and also for all variants together, adjusting for age, gender and the first five genetic principal components. A weighted linear kernel was used with each variant weighted by *Beta*(*MAF*,1,25), the beta distribution density function. After testing each region within each of the five sub-populations, we further adopted the Fisher’s combined probability test to integrate the *p*-values from sub-populations for an overall *p*-value.

### Cross-Check With Expression Quantitative Trait Loci (eQTL) Database

The majority of variants identified by existing GWASs were located in the non-coding regions of the genome, and were therefore likely to be involved in gene regulation. One hypothesis is that that causal genetic variants for complex diseases may function through regulating the expression level of genes within specific tissues. To prioritize our findings, we further examined if the identified genes harbor any known expression quantitative trait locus (eQTL) in the database. We used the Genotype-Tissue Expression (GTEx) database (GTEx Consortium, 2013) for cross checking. There are two main types of skin tissues available in the GTEx, including sun-exposed skin at lower leg and sun-unexposed skin in suprapubic region. We summarized the number of eQTLs located within each identified region for either of skin tissue types.

## Results

### Study Population

Our study included a total of 1,710 cSCC cases and 24,304 controls, partitioned into five sub-populations based on genotyping platforms. The number of subjects and their characteristics by each population is summarized in Table 1. The case-control ratios ranged from 1:6 to 1:31 across five populations. Gender was statistically different between cases and controls in four populations (*p* < 0.05), which was consist with the fact that the incidence rate was higher in men than in women (Karagas et al., 1999; Nguyen et al., 2014). Age, a well-established risk factor, was associated with cSCC in all populations (*p* < 0.001).

**TABLE 1:** Study population characteristics and number of regions tested in each population.

Population		n (%)	Male		Age	
		
			n (%)	*p*-value^*a*^	Mean (SD)	*p*-value^*a*^
**Affy (*n* = 5,533)**						
	Cases	340 (6.1)	166 (48.8)	0.004	50.34 (9.53)	<0.001
	Controls	5193 (93.9)	2118 (40.8)		48.10 (9.48)	
**Illumina (*n* = 3,314)**						
	Cases	200 (6.0)	63 (31.5)	0.002	48.25 (8.70)	<0.001
	Controls	3114 (94.0)	683 (21.9)		43.72 (8.71)	
**Omni (*n* = 5,354)**						
	Cases	737 (14.0)	281 (38.1)	0.310	48.51 (9.52)	<0.001
	Controls	4517 (86.0)	1631 (36.1)		46.90 (8.90)	
**Onco (*n* = 5,267)**						
	Cases	226 (4.3)	94 (41.6)	<0.001	47.80 (9.77)	<0.001
	Controls	5041 (95.7)	866 (17.2)		41.01 (8.87)	
**HumanCore (*n* = 6,646)**						
	Cases	207 (3.1)	102 (49.3)	<0.001	48.40 (10.24)	<0.001
	Controls	6439 (96.9)	1262 (19.6)		40.96 (9.54)	

**^*a*^p-value by two-sample t-test for age and by Chi-square test for gender.**

### Replication of GWAS Identified SNPs Using Single-Locus Testing

**FIGURE 1:**
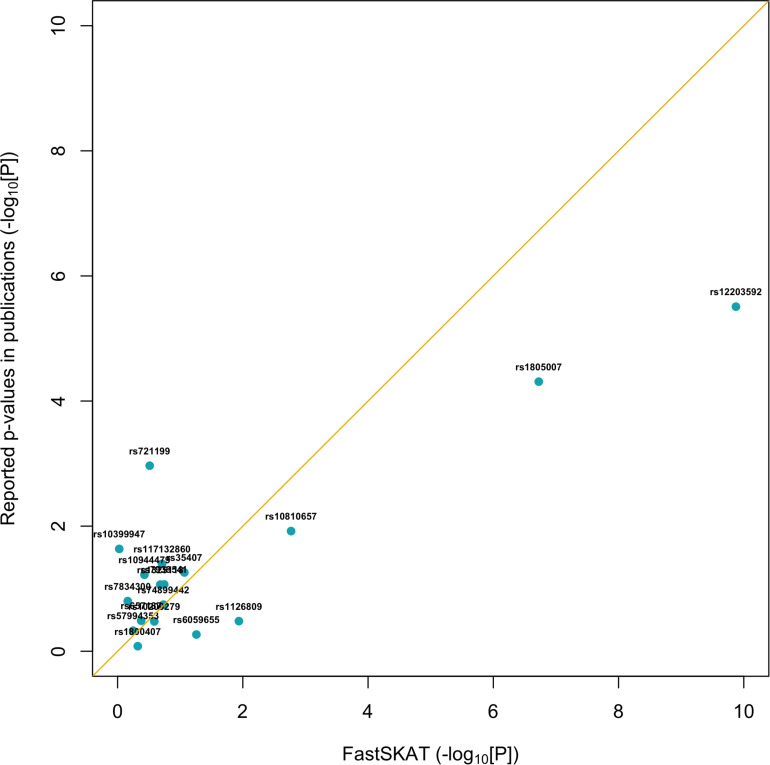
Replication of 18 GWAS identified SNPs using fastSKAT. The *p*-values of fastSKAT were based on Fisher’s method combining its testing *p*-values from the same NHS and HPFS populations used in previous publications.

For a total of 18 SNPs identified by previous GWASs, we used fastSKAT to test each variant for association with the disease outcome and compared the testing *p*-values with those reported in previous publications. The comparison is presented in Figure 1 and summarized in Table 2. We found that the Fisher’s *p*-values combining fastSKAT results of multiple populations were highly correlated with the reported *p-*values in previous publications. The Fisher’s combined *p*-values tend to be smaller, especially for variants with relatively small testing *p*-values (e.g., <0.01), leading to a higher level of statistical significance for the association. The results suggested that testing with fastSKAT in each population and combining with Fisher’s combined probability test was able to reliably identify the gene-disease association with improved statistical power.

**TABLE 2:** Comparison of *p*-values for 18 SNPs identified by published GWASs and computed by fastSKAT.

Publication	SNP	Chro	Gene^*c*^	*p*-value in paper^*d*^	*p*-value by fastSKAT^*e*^
Sarin et al., 2020^*a*^	rs10399947	1	*ARNT–[]–SETDB1*	2.31 × 10^–2^	9.41 × 10^–1^
	rs10200279	2	*ALS2CR12*	3.34 × 10^–1^	2.59 × 10^–1^
	rs10944479	6	*BACH2*	5.99 × 10^–2^	3.73 × 10^–1^
	rs7834300	8	*TRPS1*	1.58 × 10^–1^	6.89 × 10^–1^
	rs1325118	9	*[]–TYRP1*	8.60 × 10^–2^	2.08 × 10^–1^
	rs7939541	11	*ZNF143–[]–WEE1*	8.55 × 10^–2^	1.80 × 10^–1^
	rs657187	12	*KRT6A–[]–KRT5*	3.25 × 10^–1^	4.20 × 10^–1^
	rs721199	12	*HAL*	1.08 × 10^–3^	3.07 × 10^–1^
Chahal et al., 2016^*b*^	rs12203592	6	*IRF4*	3.10 × 10^–6^	1.33 × 10^–10^
	rs1805007	16	*MC1R*	4.90 × 10^–5^	1.88 × 10^–7^
	rs35407	5	*SLC45A2*	5.50 × 10^–2^	8.56 × 10^–2^
	rs1126809	11	*TYR*	3.30 × 10^–1^	1.15 × 10^–2^
	rs6059655	20	*RALY-ASIP*	5.40 × 10^–1^	5.51 × 10^–2^
	rs1800407	15	*OCA2*	8.30 × 10^–1^	4.76 × 10^–1^
	rs57994353	9	*SEC16A*	4.70 × 10^–1^	5.65 × 10^–1^
	rs10810657	9	*BNC2, CNTLN*	1.20 × 10^–2^	1.70 × 10^–3^
	rs74899442	11	*CADM1, BUD13*	1.80 × 10^–1^	1.85 × 10^–1^
	rs117132860	7	*AHR*	4.00 × 10^–2^	1.94 × 10^–1^

*^*a*^Sarin et al. (2020). Genome-wide meta-analysis identifies eight new susceptibility loci for cutaneous squamous cell carcinoma. Nat Commun 11, 820.*

*^*b*^Chahal et al. (2016). Genome-wide association study identifies novel susceptibility loci for cutaneous squamous cell carcinoma. Nat Commun 7, 12048.*

*^*c*^The format gene–[]– indicates SNPs are located within intergenic regions.*

*^*d*^p-values reported in previous publications using either three or five NHS/HPFS populations.*

*^*e*^p-values of Fisher’s method combining fastSKAT p-values from NHS/HPFS populations used in previous publications.*

### Region-Based Association Test

**FIGURE 2:**
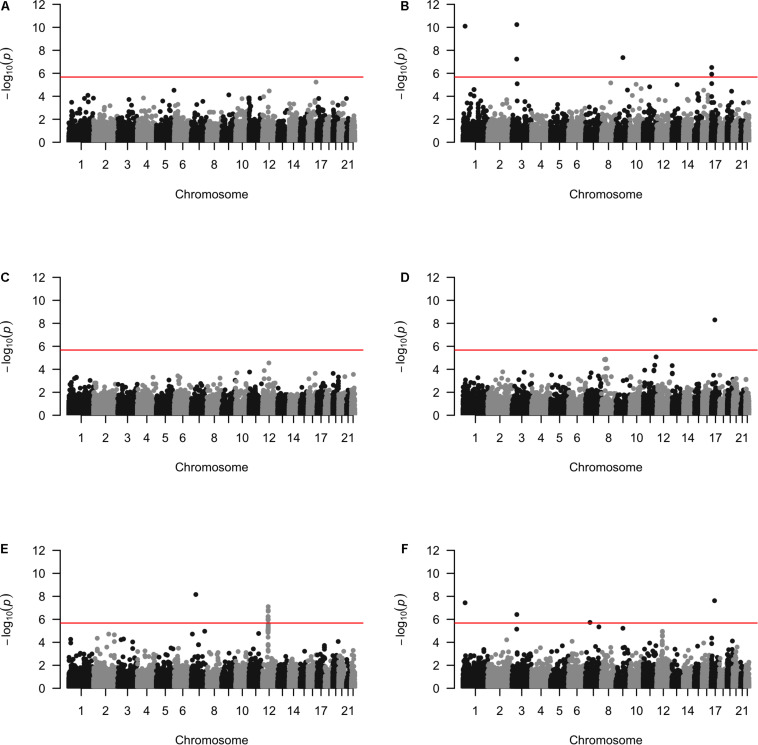
The Manhattan plots by rare variants analysis in each population **(A)** Affymetrix. **(B)** Illumina. **(C)** OmniExpress. **(D)** OncoArray. **(E)** HumanCore. **(F)** Fisher.

**FIGURE 3:**
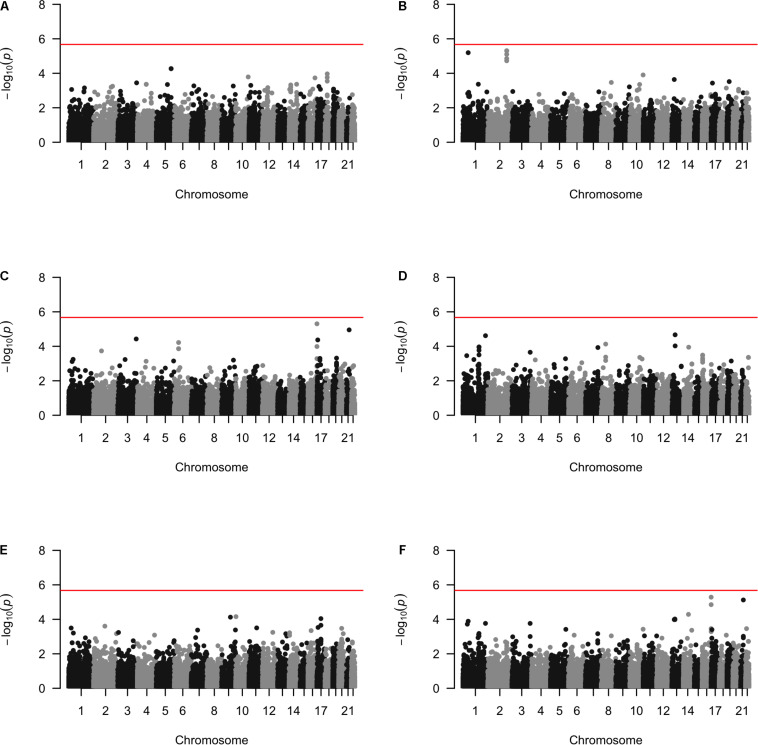
The Manhattan plots by common variants analysis in each population **(A)** Affymetrix. **(B)** Illumina. **(C)** OmniExpress. **(D)** OncoArray. **(E)** HumanCore. **(F)** Fisher.

**FIGURE 4:**
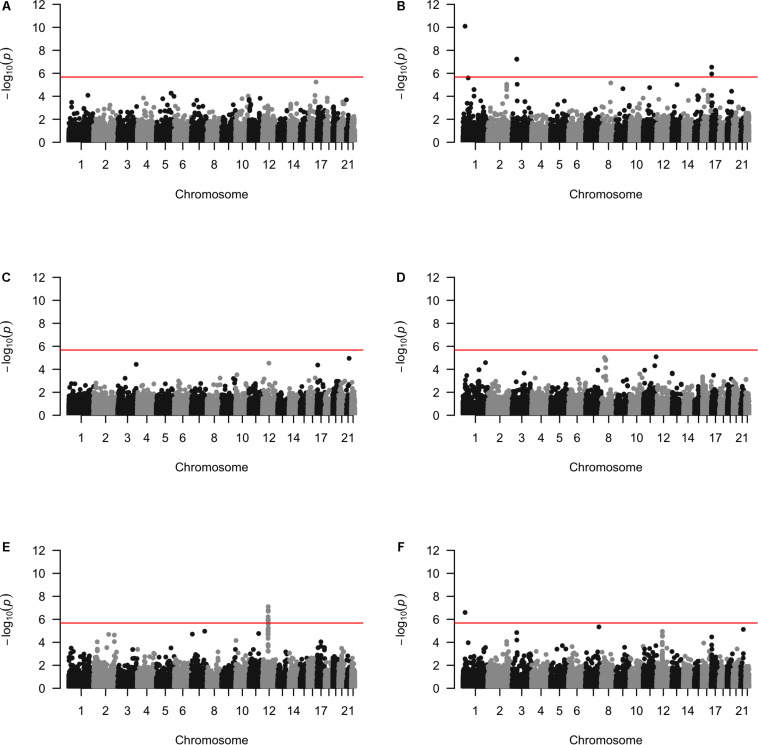
The Manhattan plots by all variants analysis in each population **(A)** Affymetrix. **(B)** Illumina. **(C)** OmniExpress. **(D)** OncoArray. **(E)** HumanCore. **(F)** Fisher.

Approximately 23,000 candidate regions were extracted and tested in each population. The numbers differed slightly across populations and was listed in Table 3. For each candidate region, the rare variants, common variants and all variants were tested separately for association with cSCC outcome using fastSKAT. The distribution of testing *p*-values were examined against a uniform distribution via quantile-quantile plots (Supplementary Figures 1–3 for rare, common and all variants, respectively). The genomic inflation factors ranged between 0.974 and 1.07, suggesting well-controlled type I error rates. The Manhattan plots based on fastSKAT and Fisher’s method are provided in Figures 2–4.

**TABLE 3:** Total number of regions and genetic variants tested in each population.

Population	Rare variants				Common variants				All variants			
			
	# of regions	# of SNPs in regions		Significance level^*a*^	# of regions	# of SNPs in regions		Significance level^*a*^	# of regions	# of SNPs in regions		Significance level^*a*^
								
		Range	Median			Range	Median			Range	Median	
Affy	23,566	2–26,354	131	2.12 × 10^–6^	23,552	2–13,667	79	2.12 × 10^–6^	23,675	2–40,021	210	2.11 × 10^–6^
Illumina	23,565	2–26,485	131	2.12 × 10^–6^	23,518	2–13,673	80	2.13 × 10^–6^	23,661	2–40,158	211	2.11 × 10^–6^
Omni	23,645	2–27,077	157	2.11 × 10^–6^	23,619	2–13,700	80	2.12 × 10^–6^	23,729	2–40,777	230	2.11 × 10^–6^
Onco	23,546	2–24,220	120	2.12 × 10^–6^	23,540	2–13,655	79	2.12 × 10^–6^	23,673	2–37,875	198	2.11 × 10^–6^
HumanCore	23,734	2–18,549	109	2.11 × 10^–6^	23,699	2–13,648	79	2.11 × 10^–6^	23,823	2–32,197	214	2.10 × 10^–6^
Fisher	23,844	–	–	2.10 × 10^–6^	23,803			2.10 × 10^–6^	23,897	–	–	2.09 × 10^–6^

**^*a*^Bonferroni adjusted significance level.**

A total of four genomic regions were identified by Fisher’s combined probability test at the Bonferroni adjusted significance level. The genomic regions and their testing *p*-values are listed in Table 4. Four regions were identified via rare variants association, and one of them was also identified via all variants analysis. No regions reached statistical significance after Bonferroni adjustment via common variants analysis. While the overall significant association was largely driven by one particular population for most of these regions, the association for one region was replicated by one additional population in the study. In particular, a region (gene *SLFN12*) was located on chromosome 17, BP: 33,737,940–33,760,195. The rare variant association test achieved statistical significance after Bonferroni correction (*p* = 2.40 × 10^–8^). The association was highly significant in “OncoArray” population (*p* = 5.05 × 10^–9^) and was replicated in “HumanCore” population (*p* = 3.73 × 10^–3^).

**TABLE 4:** Regions identified by Fisher’s combined probability test after Bonferroni adjustment.

	Chro	Regions	Gene	*p*-value					
	
				Affy	Illumina	Omni	Onco	Human Core	Fisher
Rare variants analysis	1	21,069,170–21,113,181	*HP1BP1*	7.90 × 10^–1^	**7.97 × 10**^–^**^11^**	8.47 × 10^–1^	3.62 × 10^–1^	6.99 × 10^–2^	**3.65 × 10**^–^**^8^**
	3	49,506,135–49,573,051	*DAG1*	8.62 × 10^–1^	**5.80 × 10**^–^**^11^**	8.30 × 10^–1^	7.00 × 10^–1^	7.32 × 10^–1^	**3.83 × 10**^–^**^7^**
	7	45,763,385–45,808,617	*SEPT7P2*	5.35 × 10^–1^	7.72 × 10^–1^	1.07 × 10^–1^	4.56 × 10^–1^	**6.94 × 10**^–^**^9^**	**1.86 × 10**^–^**^6^**
	17	33,737,940–33,760,195	*SLFN12*	1.64 × 10^–1^	6.11 × 10^–1^	4.38 × 10^–1^	**5.05 × 10**^–^**^9^**	**3.73 × 10**^–^**^3^**	**2.40 × 10**^–^**^8^**
All variants analysis	1	21,069,170–21,113,181	*HP1BP1*	8.29 × 10^–1^	**8.03 × 10**^–^**^11^**	5.86 × 10^–1^	9.51 × 10^–1^	3.52 × 10^–1^	**2.54 × 10**^–^**^7^**

*Bold values indicate significant association after Bonferroni adjustment in the discovery phase or nominal significant association in the replication phase.*

We further looked into the significant findings within each population. In Table 5, we summarized the regions that were identified in a particular population by both rare variants and all variants association test. In Table 6, we summarized the regions that were identified by rare variants association test only. The *p*-values computed in five populations for these regions were summarized in Supplementary Tables 1, 2. In particular, the results suggested that multiple gene regions on chromosome 12 and chromosomes 17 were identified for association with the disease outcome. For example, two regions close to each other on chromosome 17 (gene *LOC101928000*, BP: 5,015,229–5,017,677 and gene *USP6*, BP: 5,019,732–5,078,326) were identified for both rare and all variants association. A different region on chromosome 17 was identified for rare variants association. While the underlying genetic mechanism and causal SNPs were not clear, we think the rare variants association test may provide findings that are complementary to existing GWAS that usually are limited to relatively common variants. For common variants analysis, we were not able to identify any regions after Bonferroni adjustment. In Table 7, we summarized regions with suggestive significance (i.e., 10^–5^) in a particular population. In particular, the association for region *SPATA2L* was marginally significant in “OmniExpress” and was also nominally significant in both “Illumina” and “OncoArray.”

**TABLE 5:** Regions identified by both rare and all variants analysis in a particular population after Bonferroni adjustment.

Population	Chro	Regions	Gene	Rare variants analysis			All variants analysis		
					
				*p*-value in this population	Fisher’s *p*-value	# of SNPs in region	*p*-value in this population	Fisher’s *p*-value	#of SNPs in region
Illumina	1	21,069,170–21,113,181	*HP1BP3*	7.97 × 10^–11^	3.65 × 10^–8^	224	8.03 × 10^–11^	2.54 × 10^–7^	296
	3	48,445,260−48,471,460	*PLXNB1*	5.82 × 10^–8^	7.17 × 10^–6^	155	5.82 × 10^–8^	1.43 × 10^–5^	187
	3	49,506,135–49,573,051	*DAG1*	5.80 × 10^–11^	3.83 × 10^–7^	169	5.99 × 10^–8^	6.37 × 10^–5^	304
	17	5,015,229–5,017,677	*LOC101928000*	1.20 × 10^–6^	4.25 × 10^–5^	78	1.14 × 10^–6^	1.72 × 10^–4^	119
	17	5,019,732–5,078,326	*USP6*	3.11 × 10^–7^	1.31 × 10^–4^	253	2.92 × 10^–7^	3.43 × 10^–5^	406
Human Core	12	56,512,003–56,516,280	*ZC3H10*	9.95 × 10^–7^	1.37 × 10^–4^	54	1.05 × 10^–6^	1.16 × 10^–4^	71
	12	56,521,985–56,538,460	*ESYT1*	1.14 × 10^–6^	1.68 × 10^–4^	102	1.16 × 10^–6^	1.66 × 10^–4^	122
	12	56,546,203–56,551,771	*MYL6B*	6.04 × 10^–7^	7.77 × 10^–5^	61	6.04 × 10^–7^	9.85 × 10^–5^	76
	12	56,660,641–56,664,750	*COQ10A*	5.68 × 10^–7^	9.10 × 10^–5^	27	1.38 × 10^–6^	5.74 × 10^–4^	53
	12	57,623,355–57,628,718	*SHMT2*	1.57 × 10^–7^	2.49 × 10^–5^	70	1.57 × 10^–7^	2.49 × 10^–5^	86
	12	57,628,685–57,634,475	*NDUFA4L2*	1.90 × 10^–7^	2.81 × 10^–5^	52	1.90 × 10^–7^	2.81 × 10^–5^	66
	12	57,637,237–57,644,976	*STAC3*	7.88 × 10^–8^	1.23 × 10^–5^	55	7.88 × 10^–8^	1.23 × 10^–5^	70
	12	57,647,546–57,824,788	*R3HDM2*	1.96 × 10^–7^	1.10 × 10^–5^	501	1.96 × 10^–7^	1.11 × 10^–5^	729
	12	57,828,467–57,845,845	*INHBC*	1.06 × 10^–6^	2.94 × 10^–5^	85	1.06 × 10^–6^	2.94 × 10^–5^	133

**TABLE 6:** Regions identified by rare variants analysis in a particular population after Bonferroni adjustment.

Population	Chro	Regions	Gene	Rare variants analysis		
				
				*p*-value in this population	Fisher’s *p*-value	# of SNPs in region
Illumina	9	71,650,478–71,715,094	*FXN*	4.32 × 10^–8^	6.01 × 10^–6^	394
Onco	17	33,737,940–33,760,195	*SLFN12*	5.05 × 10^–9^	2.40 × 10^–8^	154
HumanCore	7	45,763,385–45,808,617	*SEPT7P2*	6.94 × 10^–9^	1.86 × 10^–6^	97
HumanCore	12	56,631,590–56,652,143	*ANKRD52*	9.60 × 10^–7^	1.50 × 10^–4^	49

**TABLE 7:** Regions reaching suggestive significance level of 10^–5^ by common variants analysis.

Identification	Chro	Regions	Gene	*p*-values in each population					
				
platform									
				Affy	Illumina	Omni	Onco	Human core	Fisher
Illumina	1	52,254,865–52,344,609	*NRDC, MIR761*	2.95 × 10^–1^	**6.39 × 10**^–^**^6^**	2.50 × 10^–1^	1.13 × 10^–1^	4.91 × 10^–1^	**1.29 × 10**^–^**^4^**
	2	190,627,505–190,630,282	*OSGEPL1-AS1*	3.97 × 10^–1^	**7.95 × 10**^–^**^6^**	8.37 × 10^–1^	8.93 × 10^–1^	8.73 × 10^–1^	**3.50 × 10**^–^**^3^**
	2	190,634,992–190,649,097	*ORMDL1*	4.16 × 10^–1^	**4.94 × 10**^–^**^6^**	7.25 × 10^–1^	9.57 × 10^–1^	8.96 × 10^–1^	**2.47 × 10**^–^**^3^**
	2	190,648,810–190,742,355	*PMS1*	4.15 × 10^–1^	**4.93 × 10**^–^**^6^**	7.25 × 10^–1^	9.57 × 10^–1^	8.96 × 10^–1^	**2.47 × 10**^–^**^3^**
Omni	16	89,762,764–89,768,121	*SPATA2L*	7.03 × 10^–1^	**2.56 × 10**^–^**^2^**	**4.96 × 10**^–^**^6^**	**2.77 × 10**^–^**^2^**	1.96 × 10^–1^	**5.19 × 10**^–^**^6^**
Fisher	21	42,513,426–42,519,991	*LINC00323*	5.21 × 10^–1^	**7.41 × 10**^–^**^3^**	**1.11 × 10**^–^**^5^**	3.02 × 10^–1^	5.87 × 10^–2^	**7.54 × 10**^–^**^6^**

*No regions were genome-wide significant after Bonferroni adjustment.*

*Bold values indicate suggestive association in the discovery phase or nominal significant association in the replication phase.*

### Cross-Check With Expression Quantitative Trait Loci (eQTL) Database

To provide additional insights on the possible involvement of these identified regions in regulating gene expression, we summarized the number of known eQTLs within each region (Table 8). Most of those loci (15 out of 18) included at least one eQTL either in not-sun-exposed or sun-exposed skin tissues. Among 24,279 regions being tested, a total of 16,534 contained at least one eQTL in the GTEx database. To evaluate the overrepresentation of eQTL in the identified region, we calculated an exact *p*-value using a hyper-genomic distribution as:

**TABLE 8:** Number of eQTLs located within identified regions in skin tissues exposed or not exposed to sun.

Population	Chro	Regions	Gene	Number of eQTLs within region	
				
				Skin not exposed to sun	Skin exposed to sun
Illumina	1	21,069,170–21,113,181	*HP1BP3*	0	0
	3	48,445,260–48,471,460	*PLXNB1*	2	2
	3	49,506,135–49,573,051	*DAG1*	3	3
	17	5,015,229–5,017,677	*LOC101928000*	0	2
	17	5,019,732–5,078,326	*USP6*	1	1
HumanCore	12	56,512,003–56,516,280	*ZC3H10*	0	1
	12	56,521,985–56,538,460	*ESYT1*	2	1
	12	56,546,203–56,551,771	*MYL6B*	2	0
	12	56,660,641–56,664,750	*COQ10A*	2	4
	12	57,623,355–57,628,718	*SHMT2*	2	0
	12	57,628,685–57,634,475	*NDUFA4L2*	0	0
	12	57,637,237–57,644,976	*STAC3*	0	2
	12	576,47,546–57,824,788	*R3HDM2*	2	4
	12	57,828,467–57,845,845	*INHBC*	0	0
Illumina	9	71,650,478–71,715,094	*FXN*	1	2
Onco	17	33,737,940–33,760,195	*SLFN12*	3	4
HumanCore	7	45,763,385–45,808,617	*SEPT7P2*	3	1
HumanCore	12	56,631,590–56,652,143	*ANKRD52*	3	3

$$p_{v\,a\,l}=\sum_{k=15}^{k=18}\frac{\left(\begin{array}{c} 16,534\\ k \end{array}\right)\,\left(\begin{array}{c} 24,279-16,534\\ 18-k \end{array}\right)}{\left(\begin{array}{c} 24,279\\ 16,534 \end{array}\right)}=0.126$$

It is also worthwhile to note that most of existing studies of eQTL were also based on single-locus association test between each genetic variants and gene expression data. Though the *p*-value was not statistically significant at 0.05 level, the large proportion of identified regions harboring known eQTL suggested their possible involvement of gene expression within skin tissues.

## Discussion

In this study, we identified 18 cSCC-associated genomic regions using gene-based fastSKAT method. One region (i.e., *SLFN12*) was statistically significant in one population and replicated in another population. The eQTL analysis further supported the possible biological contribution of those regions to the genetic susceptibility of cSCC. The replication of previous GWAS-identified SNPs also demonstrated the reliability of fastSKAT in identifying susceptibility loci with improved statistical power. To our knowledge, our study is among the first ones to investigate the region-based association for cSCC on a genome-wide level.

As an effective and powerful tool, GWAS has been commonly used to investigate the genetic architecture of complex diseases, including squamous cell carcinoma. The goal of our study is to provide a complementary strategy to address a few limitations of the GWAS, especially to evaluate the rare variants with low frequencies in the populations. In our study, although the total sample size was relatively large (∼26K), the number of cases were relatively small in each sub-population (<800). In such a situation, the single-locus-based GWAS is expected to be under-powered to identify rare variants (Tong et al., 2011; Mo et al., 2015). In addition, the highly unbalanced numbers of cases and controls may also present additional challenge to both conventional GWAS and rare-variants association tests. Recent studies have suggested that the number of cases and case to control ratio may both have an impact on the statistical power and type I errors, especially under large control group scenarios (Zhang et al., 2019). It was also found that SKAT can reach reasonably high power with well-controlled type I error if the number of cases is larger than 200. In our study, the number of cases ranged between ∼200 and 700 across five subpopulations, and the results appeared to be consistent with previous studies. The QQ-plot and estimated genomic inflation factors suggested well-controlled type I errors. While we expect the statistical power will improve with additional cases, the current results also suggested that region-based association test was able to identify genomic regions though rare variants association.

A number of gene units were identified to harbor genetic variants that may contribute to the susceptibility of cSCC. One gene was identified with replicated association in two sub-populations. Gene *SLFN12*, or Schlafen family member 12, belongs to a group of genes mediating growth-inhibition as cell cycle regulators (Katsoulidis et al., 2010). Many studies have found that *SLFN12* played a key role in generating anti-tumor effects triggered by certain drugs or interventions (Katsoulidis et al., 2010; An et al., 2019; Lewis et al., 2019). For example, the drug Anagrelide (ANA) can only inhibits cancer cell growth when both *PED3A* and *SLFN12* are expressed.

A number of other gene units were identified to be associated with cSCC in one population without replication. However, they have been reported in the literature for involvement with cancer development. For example, the identified gene units *HP1BP1* and *SEPT7P2* have been found to be involved in cancer growth and progression (Dutta et al., 2014; Wang et al., 2019). In addition, gene *SPATA2L* have been identified to be associated with vitiligo in a recent study (Cai et al., 2021), and the inverse relationship between vitiligo and NMSC was suggested in many research (Paradisi et al., 2014; Rodrigues, 2017; Wu et al., 2018; Wen et al., 2020).

A number of other methods were also available for region-based association test. For example, we and others have proposed a generalized genetic random field (GGRF) method for testing the association between a set of variants and a disease phenotype (Li et al., 2014). The proposed GGRF is a similarity-based method. It maps subjects to a Euclidean space using on their genotypes as coordinates, so that subjects who are close to each other in space would have similar phenotype if there is a gene-phenotype association (Li et al., 2014). GGRF used a Wald-type of test statistic and may achieve improved power over SKAT under various disease scenario. However, fastSKAT used a score test and is more computationally efficient with the approximation by random projection. In this study, we have used fastSKAT for analysis and we showed in Appendix, GGRF would be equivalent to SKAT if a generalized score test is used.

Our study must be considered in the light of certain limitations. First, none of the association was consistently replicated in all populations. This is partly due to the heterogeneous nature of rare variants and their low allele frequencies across populations. Multiple rare mutations within the same gene can independently influence the disease (i.e., allelic heterogeneity), and rare variants in different genes can also be involved in related pathways underlying complex human diseases (i.e., locus heterogeneity) (McClellan and King, 2010). Second, due to the nature of gene-based analysis, it is not straightforward to ascertain the causal SNPs or estimate their effect on cSCC risk. We also have not considered intergenic variants that were not within the gene regions (Mo et al., 2015). Third, the existing findings based on region-based association have been limited. For example, the eQTL variants available in GTEx database were mainly identified via single-locus analysis. Additional functional analysis is needed to validate the identified regions in the future. Forth, we are also aware that the results are subject to the strengths and limitations of fastSKAT due to its assumptions and implementation. For example, we have used a weight function that is inversely correlated with the MAF of each variant (i.e., probability density of beta distribution, default option of fastSKAT). It is often helpful to incorporate functional annotation of the variants to upweight those with potentially stronger effect on the disease (Kumar et al., 2009; Lee et al., 2015; Quick et al., 2019). Further, extensions of SKAT, such as SKAT-O, were able to effectively combine the test statistics of SKAT and burden test (Lee et al., 2012), which may have improved power when the causal variants have the same direction of effects. We have adopted fastSKAT mainly because of the computational advantage for studies with a very large number of subjects and variants. It can also be helpful to improve the power in other scenarios when SKAT-O becomes feasible for extremely large studies. Fifth, no genomic region was identified by common variants analysis after Bonferroni adjustment. It is partly because the weight function adopted gave more weight to variants with low MAF and regions with common variants receiving less weight may not be able to identify. Furthermore, region-based test would be less powerful when there are a few susceptible loci with effects in this region and the total number of tested SNPs is large.

## Data Availability Statement

The data analyzed in this study is subject to the following licenses/restrictions: GWAS data has not been publicly available. Further information including the procedures to obtain and access data from the Nurses’ Health Studies and Health Professionals Follow-up Study is described at https://www.nurseshealthstudy.org/researchers (contact email: nhsaccess@channing.harvard.edu) and https://sites.sph.harvard.edu/hpfs/for-collaborators/. The expression quantitative trait loci (eQTL) database are openly available from the Genotype-Tissue Expression (GTEx) project at https://www.gtexportal.org/home/.

## Ethics Statement

The studies involving human participants were reviewed and approved by the institutional review boards of the Brigham and Women’s Hospital and Harvard T.H. Chan School of Public Health, and those of participating registries as required. The patients/participants provided their written informed consent to participate in this study.

## Author Contributions

MH and ML conceived and designed the analysis. JH and AQ collected the data. MH, CL, XL, AQ, JH, and ML contributed data and analysis tools and wrote the manuscript. MH, CL, and ML performed the analysis. All authors have read and approved the manuscript.

## Conflict of Interest

The authors declare that the research was conducted in the absence of any commercial or financial relationships that could be construed as a potential conflict of interest.

## Appendix

In our study, a fastSKAT method was applied to test the association between each genomic region and disease outcome. A number of other methods were also available for region-based association test. For example, we and others have proposed a generalized genetic random field (GGRF) method for testing the association between a set of variants and a disease phenotype (Li et al., 2014), and compared its performance to that of SKAT. We described below that GGRF would have similar test statistic with SKAT if a generalized score test is used for inference.

Suppose the study include a total of *N* subjects, each with *K* variants in a region and *M* covariates. Let *Y*, *G*, *X* denotes the phenotype (*N* = 1), genotype (*N* = K), and covariates (*N* = M) matrix, respectively. The GGRF adopts a conditional autoregression model as:

$$E\left(Y|Y_{-}\right)=\mu \gamma S\left(Y-\mu \right),;$$

Where the *i*-th element of *Y*_−_ denotes the phenotype of all other subjects other than *i*-th subject, μ = *f*(*X*β) is used for covariants adjustment, and *S* is a matrix for pairwise genetic similarity among *N* subjects. To test the genotype-phenotype association (*H*_0_:γ = 0), a generalized score test can be used (Liang and Zeger, 1989), so that:

$$U_{\gamma }\left(\beta ,\gamma \right)={\frac{\partial E\left(Y|Y_{-}\right)}{\partial \gamma }}^{T}\left\{Y-E\left(Y|Y_{-}\right)\right\}={\left(Y-\mu \right)}^{T}S\left\{I-\gamma S\right\}\left(Y-\mu \right)=0;$$

A generalized score statistic can thus be defined as (Boos, 1992)

$$Q=U_{\gamma }\,\left(\hat{\beta },0\right)={\left(Y-\hat{\mu }\right)}^{\prime }\,S\,\left(Y-\hat{\mu }\right);$$

where $\hat{\beta }$ is estimated under the null hypothesis that γ = 0 via a generalized linear model. The score statistic $\frac{1}{m}\,Q$ takes the same format with that of SKAT, and follows asymptotically a mixture of Chi-square distributions (Wu et al., 2011).
