# Supplementary material for: Identifying Susceptibility Loci for Cutaneous Squamous Cell Carcinoma Using a Fast Sequence Kernel Association Test
Source: Front Genet. 2021 May 10;12:657499. doi: 10.3389/fgene.2021.657499 (PMC8141858; doi:10.3389/fgene.2021.657499)
Supplement: Supplementary file 1 [file Data_Sheet_1.PDF]

Supplementary Material

1 Supplementary Figures and Tables

1.1 Supplementary Figures

Supplementary Figure 1. The Q-Q plots of the observed p-values versus expected p-values under null hypothesis of no association in rare variants analysis. **(A)** Affymetrix. **(B)** Illumina. **(C)** OmniExpress. **(D)** OncoArray. **(E)** HumanCore. **(F)** Fisher

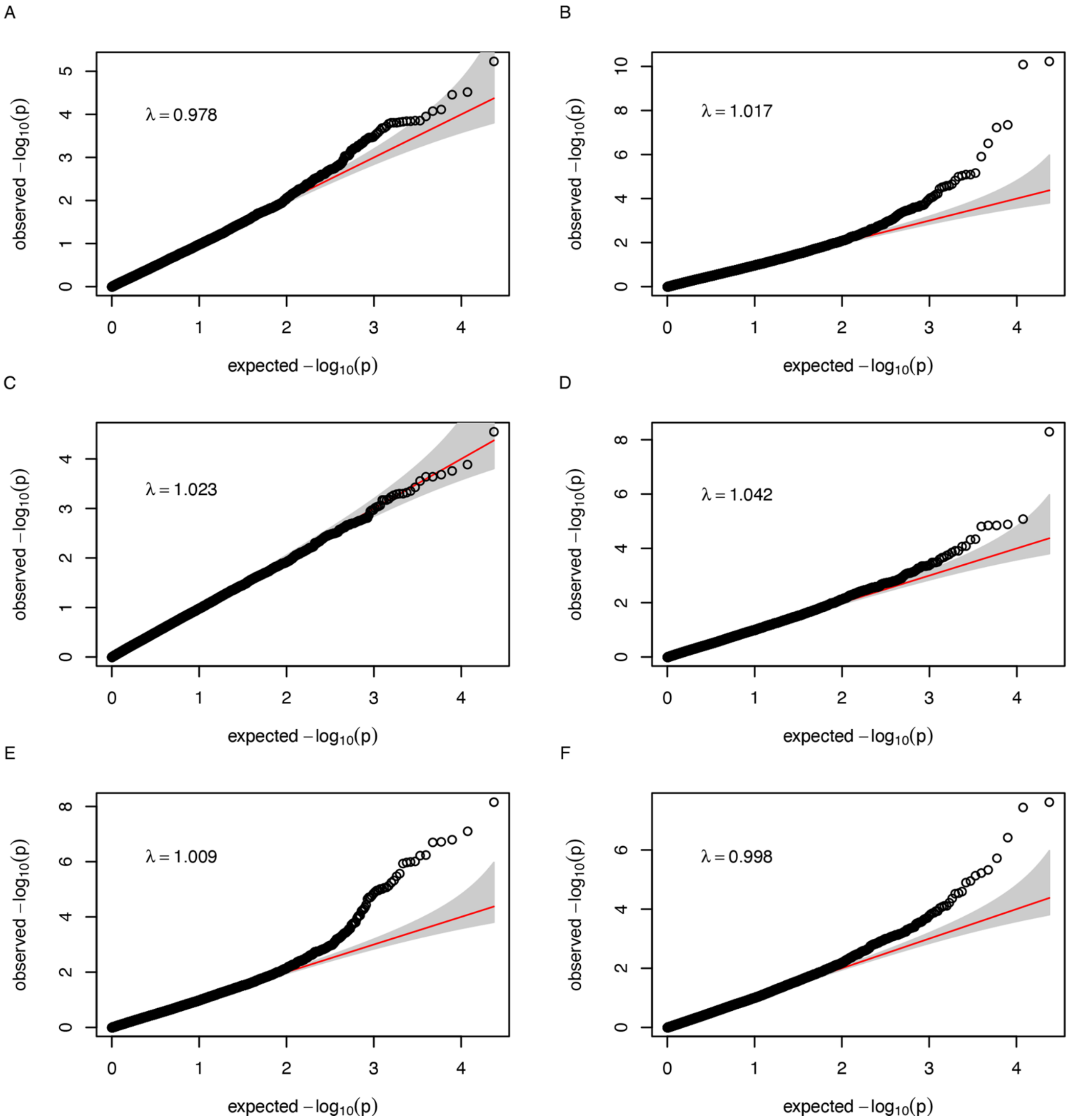

Supplementary Figure 2. The Q-Q plots of observed p-values versus expected p-values under null hypothesis of no association in common variants analysis. **(A)** Affymetrix. **(B)** Illumina. **(C)** OmniExpress. **(D)** OncoArray. **(E)** HumanCore. **(F)** Fisher

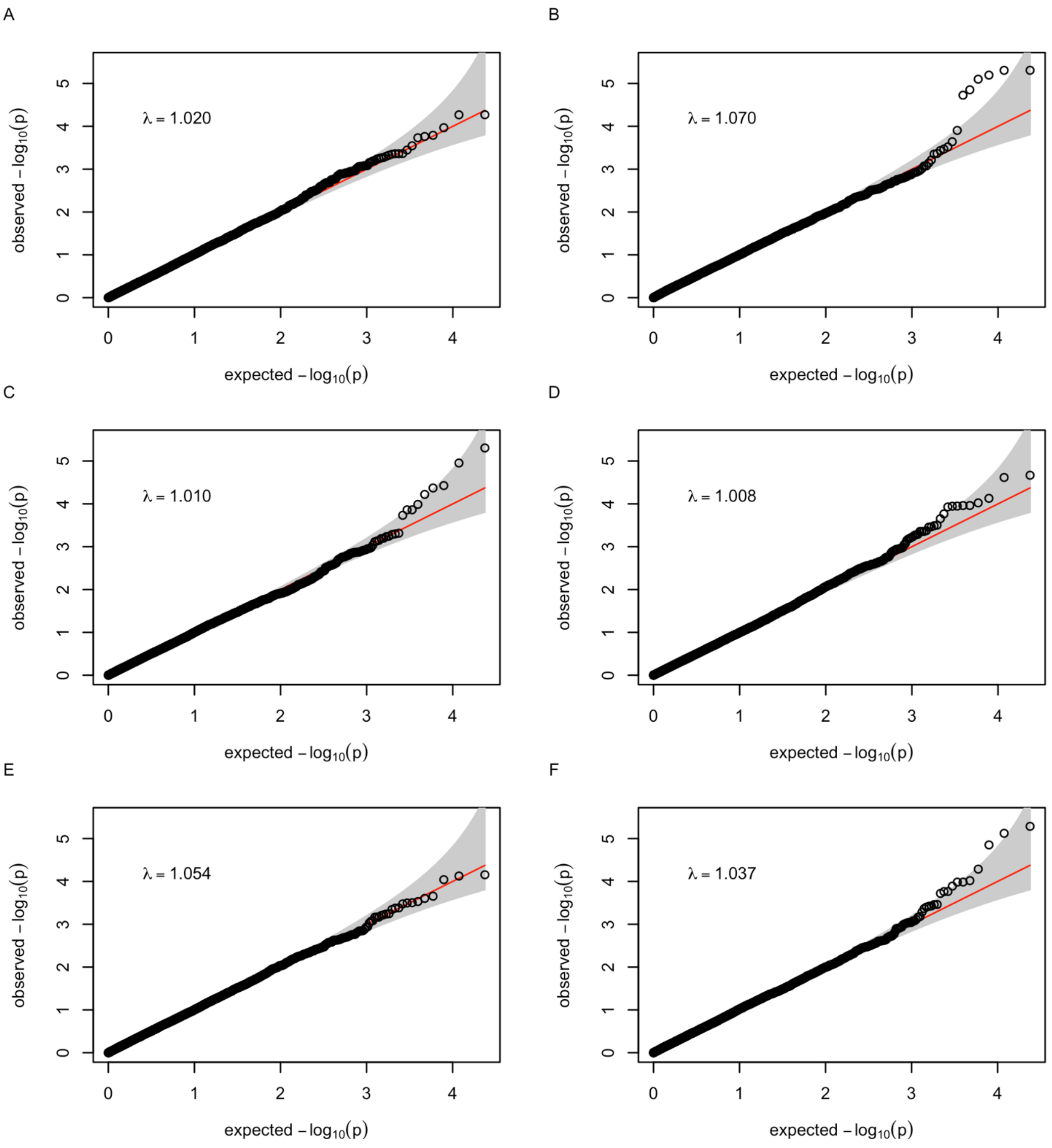

Supplementary Figure 3. The Q-Q plots of observed p-values versus expected p-values under null hypothesis of no association in all variants analysis. **(A)** Affymetrix. **(B)** Illumina. **(C)** OmniExpress. **(D)** OncoArray. **(E)** HumanCore. **(F)** Fisher

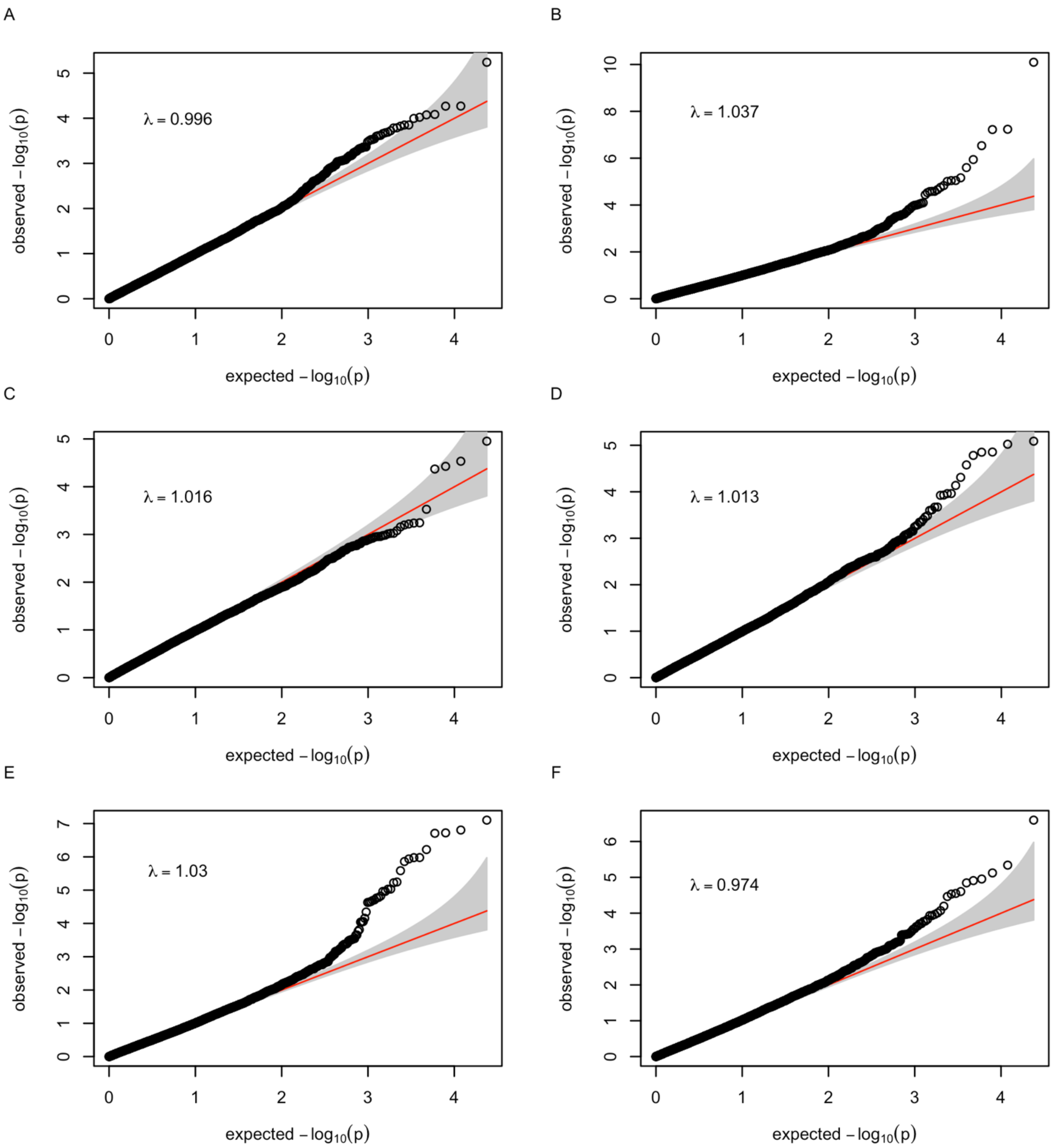

1.2 Supplementary Tables

Supplementary Table 1. Regions identified by rare variants analysis in an individual population and their p-values in all populations

Identification platform	Chro	Regions	*p*-values in each population					
			Affy	Illumina	Omni	Onco	HumanCore	Fisher
Illumina	1	21069170-21113181	8.29×10^{-1}	8.03×10^{-11}	5.87×10^{-1}	9.51×10^{-1}	3.52×10^{-1}	2.54×10^{-7}
	3	48445260-48471460	4.12×10^{-1}	5.82×10^{-8}	4.90×10^{-1}	9.07×10^{-1}	1.57×10^{-1}	1.43×10^{-5}
	3	49506135-49573051	5.57×10^{-1}	5.99×10^{-8}	3.48×10^{-1}	9.30×10^{-1}	9.94×10^{-1}	6.37×10^{-5}
	17	5015229-5017677	7.74×10^{-1}	1.14×10^{-6}	1.46×10^{-1}	5.90×10^{-1}	4.95×10^{-1}	1.72×10^{-4}
	17	5019732-5078326	6.65×10^{-1}	2.92×10^{-7}	2.51×10^{-1}	6.19×10^{-1}	1.64×10^{-1}	3.43×10^{-5}
HumanCore	12	56512003-56516280	3.11×10^{-1}	3.18×10^{-1}	5.87×10^{-1}	3.75×10^{-1}	1.05×10^{-6}	1.16×10^{-4}
	12	56521985-56538460	5.17×10^{-1}	2.76×10^{-1}	6.35×10^{-1}	3.44×10^{-1}	1.16×10^{-6}	1.66×10^{-4}
	12	56546203-56551771	4.13×10^{-1}	3.01×10^{-1}	4.59×10^{-1}	5.39×10^{-1}	6.04×10^{-7}	9.85×10^{-5}
	12	56660641-56664750	6.38×10^{-1}	6.02×10^{-1}	6.75×10^{-1}	5.04×10^{-1}	1.38×10^{-6}	5.74×10^{-4}
	12	57623355-57628718	3.74×10^{-1}	6.09×10^{-1}	3.38×10^{-1}	2.76×10^{-1}	1.57×10^{-7}	2.49×10^{-5}
	12	57628685-57634475	3.61×10^{-1}	6.39×10^{-1}	3.25×10^{-1}	2.70×10^{-1}	1.90×10^{-7}	2.81×10^{-5}
	12	57637237-57644976	3.59×10^{-1}	7.06×10^{-1}	2.73×10^{-1}	2.56×10^{-1}	7.88×10^{-8}	1.23×10^{-5}
	12	57647546-57824788	4.29×10^{-1}	4.83×10^{-1}	2.00×10^{-1}	1.52×10^{-1}	1.96×10^{-7}	1.11×10^{-5}
	12	57828467-57845845	6.44×10^{-1}	3.57×10^{-1}	1.66×10^{-1}	1.01×10^{-1}	1.06×10^{-6}	2.94×10^{-5}

Supplementary Table 2. Regions identified by all variants analysis in an individual population and their p-values in all populations

Identification platform	Chro	Regions	*p*-values in each population					
			Affy	Illumina	Omni	Onco	HumanCore	Fisher
Illumina	1	21069170-21113181	7.90×10^{-1}	7.97×10^{-11}	8.47×10^{-1}	3.62×10^{-1}	6.99×10^{-2}	3.65×10^{-8}
	3	48445260-48471460	1.50×10^{-1}	5.82×10^{-8}	4.90×10^{-1}	9.25×10^{-1}	1.81×10^{-1}	7.17×10^{-6}
	3	49506135-49573051	8.62×10^{-1}	5.80×10^{-11}	8.30×10^{-1}	7.00×10^{-1}	7.32×10^{-1}	3.83×10^{-7}
	9	71650478-71715094	8.09×10^{-1}	4.32×10^{-8}	1.15×10^{-1}	1.47×10^{-1}	9.82×10^{-1}	6.01×10^{-6}
	17	5015229-5017677	1.97×10^{-1}	1.20×10^{-6}	1.02×10^{-1}	5.69×10^{-1}	4.72×10^{-1}	4.25×10^{-5}
	17	5019732-5078326	6.21×10^{-1}	3.11×10^{-7}	3.69×10^{-1}	6.13×10^{-1}	6.09×10^{-1}	1.31×10^{-4}
Onco	17	33737940-33760195	1.64×10^{-1}	6.11×10^{-1}	4.38×10^{-1}	5.05×10^{-9}	3.73×10^{-3}	2.40×10^{-8}
HumanCore	7	45763385-45808617	5.35×10^{-1}	7.72×10^{-1}	1.07×10^{-1}	4.56×10^{-1}	6.94×10^{-9}	1.86×10^{-6}
	12	56512003-56516280	2.80×10^{-1}	5.44×10^{-1}	5.59×10^{-1}	3.33×10^{-1}	9.95×10^{-7}	1.37×10^{-4}
	12	56521985-56538460	5.09×10^{-1}	2.95×10^{-1}	6.31×10^{-1}	3.37×10^{-1}	1.14×10^{-6}	1.68×10^{-4}
	12	56546203-56551771	4.13×10^{-1}	2.45×10^{-1}	4.18×10^{-1}	5.39×10^{-1}	6.04×10^{-7}	7.77×10^{-5}
	12	56631590-56652143	2.95×10^{-1}	4.73×10^{-1}	4.93×10^{-1}	4.78×10^{-1}	9.60×10^{-7}	1.50×10^{-4}
	12	56660641-56664750	4.64×10^{-1}	4.18×10^{-1}	4.77×10^{-1}	3.20×10^{-1}	5.68×10^{-7}	9.10×10^{-5}
	12	57623355-57628718	3.74×10^{-1}	6.09×10^{-1}	3.38×10^{-1}	2.76×10^{-1}	1.57×10^{-7}	2.49×10^{-5}
	12	57628685-57634475	3.61×10^{-1}	6.39×10^{-1}	3.25×10^{-1}	2.70×10^{-1}	1.90×10^{-7}	2.81×10^{-5}
	12	57637237-57644976	3.59×10^{-1}	7.06×10^{-1}	2.73×10^{-1}	2.56×10^{-1}	7.88×10^{-8}	1.23×10^{-5}
	12	57647546-57824788	4.31×10^{-1}	4.71×10^{-1}	2.00×10^{-1}	1.53×10^{-1}	1.96×10^{-7}	1.10×10^{-5}
	12	57828467-57845845	6.44×10^{-1}	3.57×10^{-1}	1.66×10^{-1}	1.01×10^{-1}	1.06×10^{-6}	2.94×10^{-5}
